# Host Cell Binding Mediated by *Leptospira interrogans* Adhesins

**DOI:** 10.3390/ijms232415550

**Published:** 2022-12-08

**Authors:** Maria Beatriz Takahashi, Aline Florencio Teixeira, Ana Lucia Tabet Oller Nascimento

**Affiliations:** 1 Programa de Pós-Graduação Interunidades em Biotecnologia, Instituto de Ciências Biomédicas, Universidade de São Paulo, São Paulo 05508-900, SP, Brazil; 2 Laboratório de Desenvolvimento de Vacinas, Instituto Butantan, Avenida Vital Brazil, São Paulo 05503-900, SP, Brazil

**Keywords:** *Leptospira*, leptospirosis, recombinant protein, host–pathogen interactions, cell culture, mammalian cell culture, adhesins

## Abstract

Leptospirosis is a neglected infectious disease with global impact on both humans and animals. The increase in urban development without sanitation planning is one of the main reasons for the disease spreading. The symptoms are similar to those of flu-like diseases, such as dengue, yellow fever, and malaria, which can result in a misleading clinical diagnosis. The characterization of host–pathogen interactions is important in the development of new vaccines, treatments, and diagnostics. However, the pathogenesis of leptospirosis is not well understood, and many gaps remain to be addressed. Here, we aimed to determine if *Leptospira* strains, virulent, culture-attenuated, and saprophytic, and the major outer membrane proteins OmpL37, OmpL1, LipL21, LipL41, and LipL46 are able to adhere to different endothelial, epithelial and fibroblast cell lines in vitro. We showed that virulent leptospires robustly bind to all cells compared to the culture-attenuated and saprophytic lines. The recombinant proteins exhibited certain adhesion, but only OmpL1 and LipL41 were able to bind to several cell lines, either in monolayer or in cell suspension. Blocking OmpL1 with polyclonal antibodies caused a decrease in bacterial binding to cells, contrasting with an increase observed when anti-LipL41 antibodies were used. The adhesion of OmpL1 to HMEC-1 and EA.hy926 was inhibited when cells were pre-incubated with collagen IV, suggesting that both compete for the same cell receptor. We present here for the first time the interaction of five leptospiral outer membrane proteins with several cell lines, and we conclude that LipL41 and OmpL1 may have an impact on leptospiral adhesion to mammalian cells and may mediate the colonization process in leptospiral pathogenesis.

## 1. Introduction

The elucidation of pathogenetic mechanisms of bacteria is an important step in understanding host–pathogen interactions. Adhesins such as pili, fimbriae, lipoproteins, and transmembrane proteins are among the first structures of pathogens to interact with and bind to host cells and tissues [1] The adhesion of those proteins to extracellular matrix (ECM) and cell receptors is necessary to ensure bacterial anchoring, cell invasion, and tissue colonization success [2].

Many bacterial species express various adhesins with the same function depending on infection stage, and they can impair the identification of virulent factors. Studies of *Borrelia* and *Leptospira* have described several membrane proteins with redundant function. Those proteins are able to bind to various ECM components, such as collagen IV, cellular fibronectin, laminin, and/or plasma components, e.g., plasminogen and fibrinogen [3,4,5]. The number of molecules involved in these interactions suggests that both bacterial genera have a range of strategies to maintain infection.

Despite the characterization of the interplay of several membrane proteins with host components, host–pathogen interactions among *Leptospira* spp. are still poorly understood. We showed that among the major outer membrane proteins, OmpL1 could interact with laminin, glycosaminoglycans, plasma fibronectin, and plasminogen [6,7]. OmpL37 is an elastin-, laminin-, plasma fibronectin- and fibrinogen-interacting protein [8], while LipL46 shows exclusive binding to plasminogen [9]. Recently, it was demonstrated that LipL21 and LipL41 exhibited a broad binding profile, including several ECM and plasma components [10], suggesting the involvement of these proteins in the initial infection process of leptospires.

The interaction of *Leptospira* spp. with diverse cell lines is well reported in the literature, ranging from murine microglial, epithelial, and endothelial cells to Vero cells and A31 fibroblasts [11,12,13,14,15,16,17]. In other investigations, leptospiral proteins, including LigA, LigB, OmpL1, rLIC11574, rLIC13411, rLIC12976 and rLIC10831, were assayed in monolayers of mammalian cells, but the main focus of these studies was the binding of proteins to glycosaminoglycans (GAGs) and ECM. Of the proteins studied, only OmpL1 showed significant binding to both mammalian cell lines tested [7,13,18,19,20,21].

Since *Leptospira* spp. have the ability to invade hosts and colonize different organs, bacterial interaction with several cell types could reveal new strategies involved in the initial infection process and its establishment. The identification of proteins that could mediate those interactions can be crucial for the development of vaccines, treatments, and diagnostic methods. The aim of this study was to examine the binding of *Leptospira* spp. to several endothelial, epithelial and fibroblast cell lines, focusing on the virulent capability of these bacteria, and to evaluate the potential of the outer membrane proteins OmpL37, OmpL1, LipL21, LipL41 and LipL46 to mediate adhesion to mammalian cells. 

## 2. Results

### 2.1. Evaluation of Leptospira Binding to Mammalian Cells by ELISA

**Figure 1:**
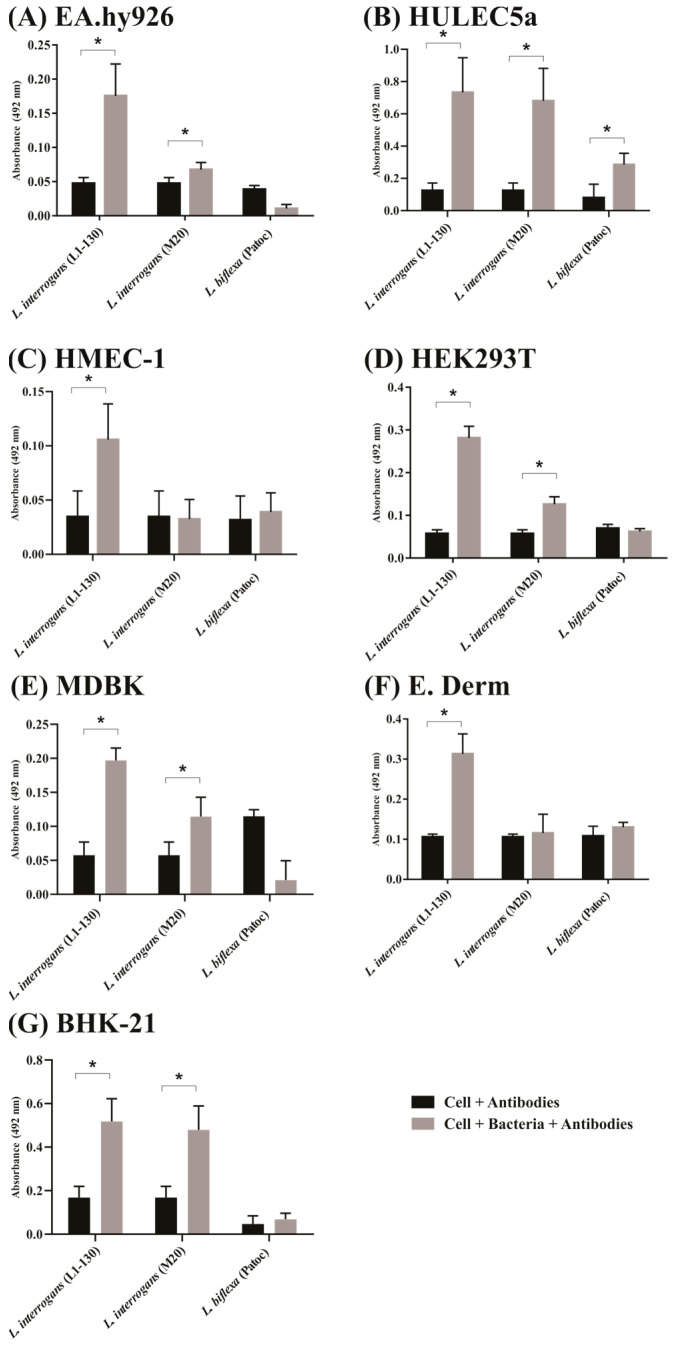
Virulent leptospires can bind to endothelial, epithelial and fibroblast cells. Mammalian cells (10^5^ cells/well) were seeded in 96-well plates treated with 0.01% poly-L-lysine and grown to confluence at 37 °C under 5% CO_2_ for 24 h. *L. interrogans* serovar Copenhageni L1-130 (virulent strain), *L. interrogans* serovar Copenhageni M20 (avirulent strain through culture passaging) and *L. biflexa* serovar Patoc (saprophytic strain) were added in cell culture media with MOI 100 (10^7^ leptospires/well) for 2 h. Wells without the addition of bacteria were used as negative control. Samples were incubated with polyclonal anti-leptospires (*L. interrogans* or *L. biflexa*, dilution of 1:1000) followed by anti-hamster IgG-peroxidase (1:5000). The binding of leptospires was compared to the negative controls by the two tailed *t*-test (*p* < 0.05) showed by *. Endothelial cells were represented by (**A**): EA.hy926, (**B**): HULEC5a and (**C**): HMEC-1. Epithelial cells were represented by (**D**): HEK293T and (**E**): MDBK. Fibroblasts were represented by (**F**): E. Derm and (**G**): BHK-21.

Although many studies have shown that leptospires are able to bind to mammalian cells in vitro [4], this is the first study to investigate leptospires binding to mammalian cells using multiple different cell lines. We determined the adhesion profile of virulent, culture-attenuated and saprophytic strains of leptospires in EA.hy926, HMEC-1 and HULEC5a endothelial cells, in HEK293T and MDBK epithelial cells, and in E. Derm and BHK-21 fibroblasts. As observed, the virulent strain *L. interrogans* serovar Copenhageni (Fiocruz L1–130) was capable of binding to all cell lines (Figure 1A–G). The binding of culture-attenuated *L. interrogans* serovar Copenhageni (M20) strain was lower than that of the virulent strain for some cell lines. When the binding to three endothelial cell lines, EA.hy926 (Figure 1A), HULEC5a (Figure 1B) and HMEC-1 (Figure 1C), was performed, we observed that only the binding to EA.hy926 cell line was reduced. HMEC-1 did not show statistical difference from the control, and the level of absorbance for HULEC5a was similar to that observed for the virulent strain. For the epithelial cells lines HEK293T (Figure 1D) and MDBK (Figure 1E), a reduction in binding was observed compared to that of the virulent strain. In relation to the fibroblast cells, for E. Derm (Figure 1F), there were no statistically significant differences. In contrast, for the BHK-21 cell line (Figure 1G), the level of binding was similar to that of the virulent strain. Saprophytic strain *L. biflexa* serovar Patoc (Patoc 1) showed binding only to the endothelial HULEC5a cell line (Figure 1B), possibly due to some sticker properties. In general, we confirmed that the bacterial adhesion results corroborate with those in the literature [11,19].

### 2.2. Evaluation of Leptospiral Membrane Protein Binding to Mammalian Cells

The proteins OmpL37, OmpL1, LipL21, LipL41, and LipL46 were successfully obtained by *E. coli* heterologous expression using the pET-SUMO vector (Figure S1). The assays with mammalian cells and the proteins were performed using monolayers and cell suspensions. The rationale to use both conditions was based on reported studies that examined whether the binding was primarily to the ECM or to mammalian cell surface receptors. Having this in mind, cells were lifted with trypsin, which detaches the cells from the ECM and also degrades proteins that are involved in cell adhesion [11].

**Figure 2:**
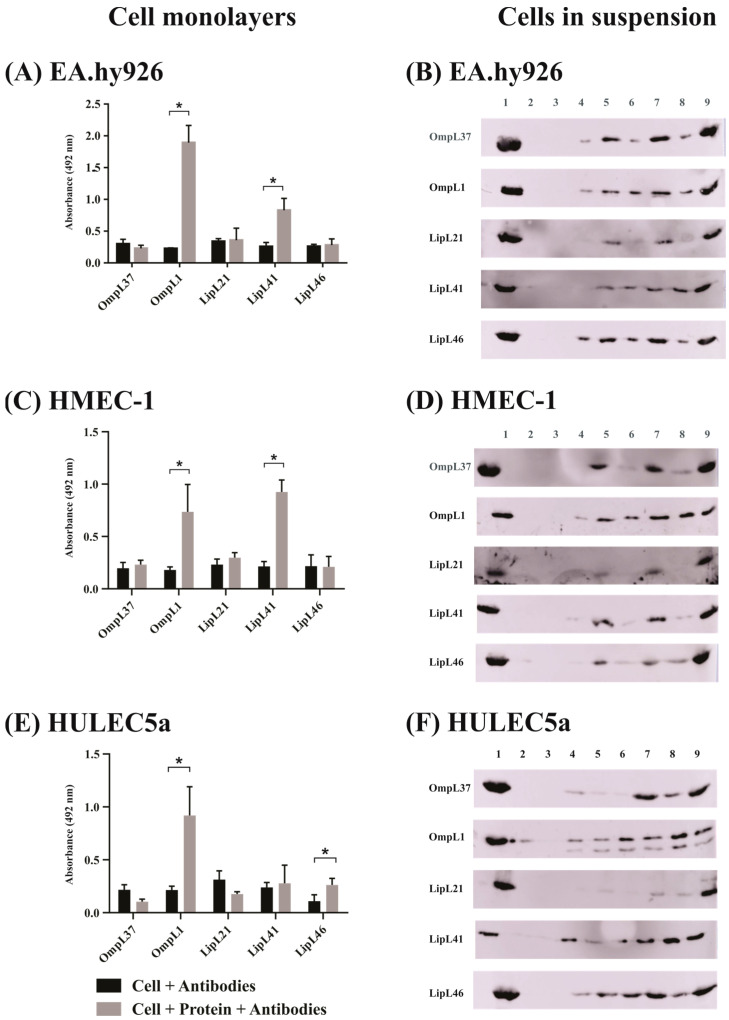
Evaluation of adhesion potential of the major outer membrane proteins of *L. interrogans* with endothelial cells. Binding of five recombinant membrane proteins, OmpL37, OmpL1, LipL21, LipL41, and LipL46, of *L. interrogans* to mammalian cells was assessed with cell monolayers (immobilized assay) and detached cells (suspension assay). (**A**,**C**,**E**)**: Cell monolayers** (**left side**). Ninety-six-well plates were seeded with 10^5^ cells/well, and cell confluence was achieved at 37 °C under 5% CO_2_ for 24 h. One microgram of each recombinant protein was resuspended in cell media and 1 µM aprotinin was added for 2 h. Wells without the addition of the proteins were used as negative control. Samples were incubated with anti-recombinant proteins (1:1000) for 1 h. Reaction was detected with anti-mouse IgG-peroxidase (1:5000). The binding of the proteins was compared to the negative controls by the two-tailed *t*-test (*p* < 0.05) showed by *. (**A**): EA.hy926, (**C**): HMEC-1 and (**E**): HULEC5a. (**B**,**D**,**F**)**: Cells in suspension** (**right side**). After cell trypsinization, 2 × 10^5^ cells were resuspended in cell media with different protein concentrations in the presence of 1 µM aprotinin for 2 h. The samples were centrifuged at 130× *g* for 10 min, and the interaction was assessed by Western blotting. (**B**): EA.hy926, (**D**): HMEC-1 and (**F**): HULEC5a. Lanes were described as 1: recombinant protein as positive control; 2–3: pellet and supernatant of negative control without addition of recombinant proteins, respectively; 4–5: pellet and supernatant with addition of 2.5 µg of protein, respectively; 6–7: pellet and supernatant with addition of 5 µg of protein, respectively; and 8–9: pellet and supernatant with addition of 10 µg of protein, respectively.

The binding of the proteins to the endothelial cells EA.hy926, HMEC-1 and HULEC5a is shown in Figure 2. As observed, OmpL1 and LipL41 showed binding to EA.hy926 and HMEC-1 cells in both monolayer and suspension assays (Figure 2A–D), while LipL46 and OmpL37 showed binding only in suspension cells (Figure 2B,D). For HULEC5a cells, OmpL1 and LipL46 showed binding in the monolayer assay (Figure 2E), and all proteins, except LipL21, showed an increase in interaction for cell suspensions (Figure 2F).

**Figure 3:**
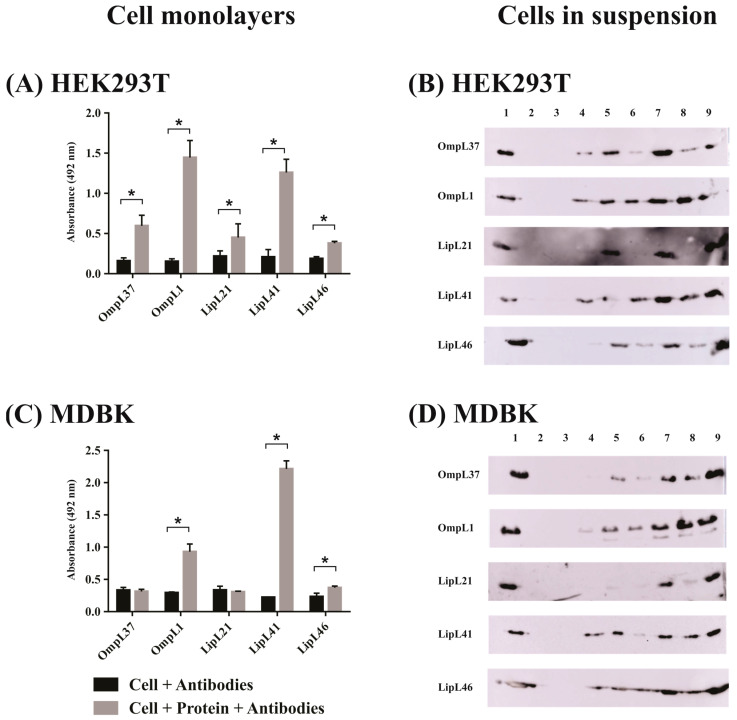
Evaluation of adhesion potential of the major outer membrane proteins of *L. interrogans* with epithelial cells. Binding of five recombinant membrane proteins, OmpL37, OmpL1, LipL21, LipL41, and LipL46, of *L. interrogans* to mammalian cells was assessed with cell monolayers (immobilized assay) and detached cells (suspension assay). (**A**,**C**)**: Cell monolayers** (**left side**). Ninety-six-well plates were seeded with 10^5^ cells/well, and cell confluence was achieved at 37 °C under 5% CO_2_ for 24 h. One microgram of each recombinant protein was resuspended in cell media, and 1 µM aprotinin was added for 2 h. Wells without the addition of the proteins were used as negative control. Samples were incubated with anti-recombinant proteins (1:1000) for 1 h. Reaction was detected with anti-mouse IgG-peroxidase (1:5000). The binding of the proteins was compared to the negative controls by the two tailed *t*-test (*p* < 0.05) showed by *. (**A**): HEK293T and (**C**): MDBK. (**B**,**D**)**: Cells in suspension** (**right side**). After cell trypsinization, 2 × 10^5^ cells were resuspended in cell media with different protein concentrations in the presence of 1 µM aprotinin for 2 h. The samples were centrifuged at 130× *g* for 10 min, and the interaction was assessed by Western blot. (**B**): HEK293T and (**D**): MDBK. Lanes were described as 1: recombinant protein as positive control; 2–3: pellet and supernatant of negative control without addition of recombinant proteins, respectively; 4–5: pellet and supernatant with addition of 2.5 µg of protein, respectively; 6–7: pellet and supernatant with addition of 5 µg of protein, respectively; and 8–9: pellet and supernatant with addition of 10 µg of protein, respectively.

**Figure 4:**
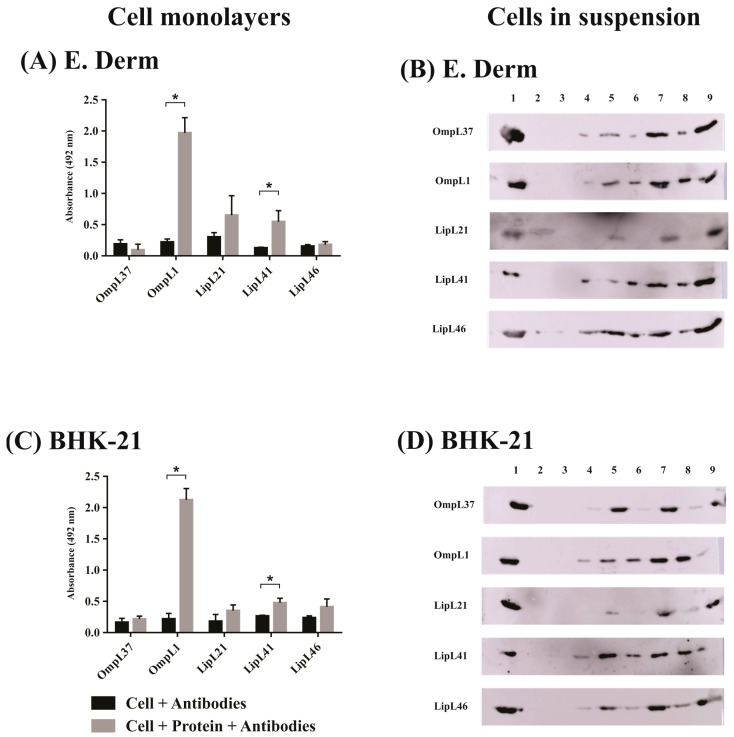
Evaluation of adhesion potential of the major outer membrane proteins from *L. interrogans* with fibroblasts. Binding of five recombinant membrane proteins, OmpL37, OmpL1, LipL21, LipL41, and LipL46, of *L. interrogans* to mammalian cells was assessed with cell monolayers (immobilized assay) and detached cells (suspension assay). (**A**,**C**)**: Cell monolayers** (**left side**). Ninety-six-well plates were seeded with 10^5^ cells/well, and cell confluence was achieved at 37 °C under 5% CO_2_ for 24 h. One microgram of each recombinant protein was resuspended in cell media, and 1 µM aprotinin was added for 2 h. Wells without the addition of the proteins were used as negative control. Samples were incubated with anti-recombinant proteins (1:1000) for 1 h. Reaction was detected with anti-mouse IgG-peroxidase (1:5000). The binding of the proteins was compared to the negative controls by the two tailed *t*-test (*p* < 0.05) showed by *. (**A**): E. Derm and C: BHK-21. (**B**,**D**)**: Cells in suspension** (**right side**). After cell trypsinization, 2 × 10^5^ cells were resuspended in cell media with different protein concentrations in the presence of 1 µM aprotinin for 2 h. The samples were centrifuged at 130× *g* for 10 min, and the interaction was assessed by Western blot. (**B**): E. Derm and (**D**): BHK-21. Lanes were described as 1: recombinant protein as positive control; 2–3: pellet and supernatant of negative control without addition of recombinant proteins, respectively; 4–5: pellet and supernatant with addition of 2.5 µg of protein, respectively; 6–7: pellet and supernatant with addition of 5 µg of protein, respectively; and 8–9: pellet and supernatant with addition of 10 µg of protein, respectively.

For the evaluation using epithelial cells (Figure 3), all proteins interacted with HEK293T human kidney cells in the monolayer assay (Figure 3A). It is interesting that both OmpL1 and LipL41 showed greater binding compared with the other proteins in monolayer (Figure 3A) and cell suspension (Figure 3B) assays. In the case of MDBK monolayer cells, only OmpL1, LipL41, and LipL46 displayed significant differences with the control groups (Figure 3C). Regarding the cells in suspension, a broad binding profile was observed (Figure 3D). The assays carried out with fibroblasts are presented in Figure 4. The results showed a similar profile for the E. Derm cells (Figure 4A,B) and BHK-21 cells (Figure 4C,D), where only OmpL1 and LipL41 interacted with monolayer cells (Figure 4A,C). When cells in suspension were assayed, a broad binding profile was also observed, showing that depending on cellular conditions, all proteins were able to interact with the cells.

When the interactions between recombinant protein and cells were evaluated in a dose response assay, we observed dose-dependent binding for all mammalian cells (Supplementary Figures S2A–F, S3 and S4). However, saturation levels were reached only for the interactions between EA.hy926 and OmpL1 (Figure S2A), HMEC-1 cell and OmpL1 (Figure S2B) and LipL41 (Figure S2C), HULEC5a cell and OmpL1 (Figure S2D); in the case of HULEC5a and LipL46 (Figure S2E) and EA.hy926 and LipL41 (Figure S2F), the saturation was not reached. Experiments with epithelial cells HEK293T showed dose-response binding with all recombinant proteins, OmpL37 (Figure S3A), OmpL1 (Figure S3B), LipL21 (Figure S3C), LipL41 (Figure S3D), and LipL46 (Figure S3E); and MDBK cell and OmpL1 (Figure S3F), LipL41 (Figure S3G), and LipL46 (Figure S3H); saturation levels were seen with fibroblasts E. Derm with OmpL1 (Figure S4A) and LipL41 (Figure S4B), and BHK-21 with OmpL1 (Figure S4C) and LipL41 (Figure S4D). Generally, the dose-dependent and saturable binding of recombinant proteins to cells indicates that these interactions are specific between ligand and cell receptor. However, as OmpL1 and LipL41 were capable of binding to three kinds of cell lines (epithelial, endothelial and fibroblasts), it suggests a higher affinity of these proteins for mammalian cell receptors. Table 1 shows the dissociation constant (K_D_) values observed from the protein–cell interactions.

### 2.3. Effect of Antiserum against the Recombinant Proteins on the Adhesion of L. interrogans to Mammalian Cells

One important aspect of the protein interactions with mammalian cells to investigate was whether these proteins had any effect on the binding of *L. interrogans* to monolayer cells. This prompted us to evaluate the influence of either the antiserum against each protein or the proteins themselves, on the binding of *L. interrogans* to mammalian cells. Most of the experiments were performed with OmpL1 and LipL41 because of their broad binding to mammalian cells. However, the binding of all proteins to HEK293T cells prompted us to examine the influence of the proteins on the interaction of *L. interrogans* and these cells.

**Figure 5:**
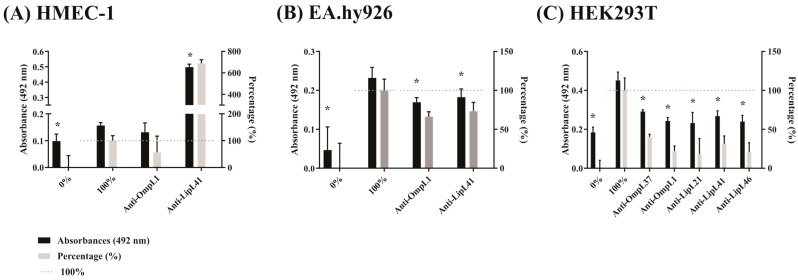
The interaction of leptospires with human cells was affected by polyclonal antibodies against the recombinant proteins. Leptospires (10^7^ leptospires/well, MOI 100) were incubated with polyclonal antibodies produced in mouse (1:100) for 1 h. Binding of bacteria was assessed in 96-well plate with cell monolayers (10^5^ cells/well) for 2 h. Wells without and with addition of untreated leptospires were used as controls. Samples were incubated with anti-LipL32 produced in hamster (1:1000). The reaction was detected with anti-hamster IgG-peroxidase. Values were represented by percentage considering the wells without and with the addition of leptospires as 0 and 100% of adhesion, respectively. The binding of the bacteria was compared to the 100% control by the two-tailed *t*-test (*p* < 0.05) showed by *. (**A**): HMEC-1, (**B**): EA.hy926, (**C**): HEK293T.

Blocking assay using polyclonal antibodies was performed to see if the interaction between leptospires and mammalian cells was impaired. Three human cell lines, HMEC-1, EA.hy926, and HEK293T, were evaluated with the virulent strain *L. interrogans* (Figure 5). For HMEC-1, when leptospires were previously incubated with polyclonal anti-OmpL1 and anti-LipL41 antibodies and then submitted to cell interaction, there was reduced adhesion for OmpL1, but this was not statistically significant. Interestingly, antiserum against the protein LipL41 increased bacterial adhesion by approximately 7 times more than the control (Figure 5A). This could suggest a particular feature of this cell line since this behavior was not observed with the other cell lines. A bridging effect could not be ruled out. The EA.hy926 cell interaction resulted in an inhibition of 26 to 33% for OmpL1 and LipL41, respectively (Figure 5B), while with HEK293T, all proteins, OmpL37, OmpL1, LipL21, LipL41, and LipL46, exhibited statistically significant inhibition of binding to these cells. We also assayed all of those interactions using a culture-attenuated strain of *L. interrogans* and EA.hy926 and HEK293T cells. In both cases, the results of inhibition were similar, as observed with the virulent strain. In general, the use of antiserum against the major outer membrane protein was effective in inhibiting bacterial adhesion to cells, showing that these proteins can be involved in cell adhesion.

### 2.4. Effect of Recombinant Proteins on the Adhesion of L. interrogans to Mammalian Cells

**Figure 6:**
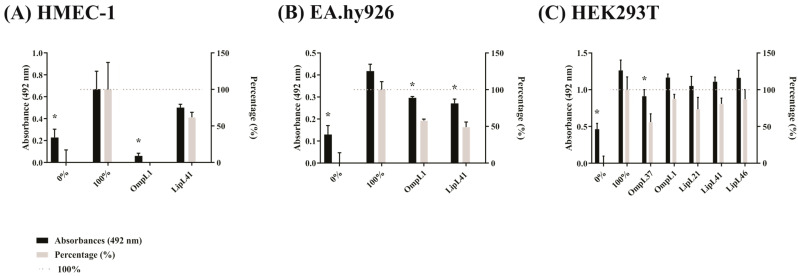
The interaction of leptospires with mammalian cells was affected by adding recombinant proteins. Binding of bacteria was assessed in 96-well plate with cell monolayers (10^5^ cells/well). Cells were incubated with recombinant proteins in saturation concentration with 1 µM aprotinin for 1 h. Wells without the addition of proteins was used for the controls. Leptospires (10^7^ leptospires/well, MOI 100) were incubated for 2 h. Wells without and with addition of leptospires were used as controls. Samples were incubated with anti-LipL32 produced in mouse, and the detection was performed with anti-mouse IgG-peroxidase. Values were represented by percentage considering the wells without and with the addition of leptospires as 0 and 100% of adhesion, respectively. The binding of the bacteria was compared to the 100% control by the two-tailed *t*-test (*p* < 0.05) showed by *. (**A**): HMEC-1, (**B**): EA.hy926, (**C**): HEK293T.

Following the same line as above, a competition assay was performed to determine the interaction between leptospires and cells in the presence of recombinant proteins. The results presented in Figure 6A show that OmpL1 was able to completely reduce bacterial adhesion when previously incubated with HMEC-1 cells. In contrast, LipL41 was not able to promote such effect and, although a partial reduction was observed, it was not statistically significant. For the EA.hy926 cell line, both OmpL1 and LipL41 were capable of competing for the same binding site with the virulent strain, showing an inhibition rate of 42 and 51%, respectively (Figure 6B). For the HEK293T cell line, neither OmpL1 nor LipL41 interfered in virulent strain adhesion to cells, and only OmpL37 was capable of interfering with binding, showing 44% inhibition (Figure 6C). The binding of the culture-attenuated strain to EA.hy926 cells was also affected by OmpL1 and LipL41, while in the case of HEK293T, only LipL41 showed a statistically significant inhibitory effect (not shown). The use of recombinant proteins to compete for the same binding site of the bacteria is an interesting approach and reveals the involvement of these proteins with host cells, mainly for OmpL1.

### 2.5. Effect of Extracellular Matrix Components on Leptospiral Protein Adhesion to Mammalian Cells

**Figure 7:**
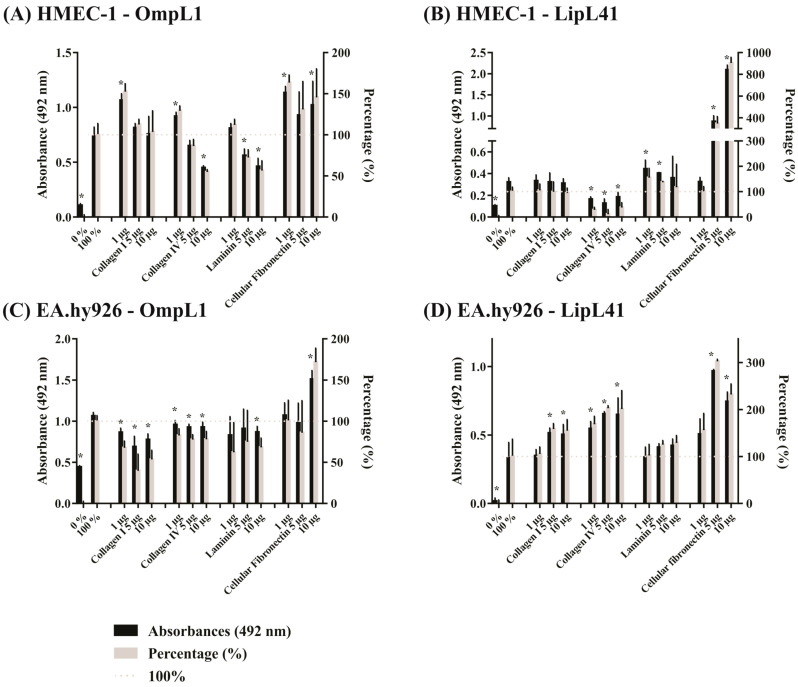
Competition assay between recombinant proteins and host components for binding to mammalian cells. Competition binding of the recombinant proteins and between the purified host components and cell receptors was assessed in 96-well plates with cell monolayers (10^5^ cells/well). Proteins were incubated with different concentrations of host components and 1 µM aprotinin in cell media for 1 h. The samples were added to wells for 2 h. Samples without and with untreated recombinant proteins were used as controls. The polyclonal antibodies against the recombinant proteins (1:1000) were added for 1 h. Detection was performed with anti-mouse IgG-peroxidase. Values were represented by percentage considering the wells without and with the addition of untreated proteins as 0 and 100% of adhesion, respectively. The binding of the proteins was compared to the 100% control by the two-tailed *t*-test (*p* < 0.05) showed by *. (**A**): HMEC-1–OmpL1, (**B**): HMEC-1–LipL41, (**C**): EA.hy926–OmpL1 and (**D**): EA.hy926–LipL41.

As several studies have shown that leptospires interact with ECM components via their outer membrane proteins [6,10,22], we examined host components as competitors of recombinant protein binding to cells. Accordingly, OmpL1 and LipL41 were evaluated after interaction with four components: collagen I, collagen IV, laminin and cellular fibronectin. As observed in Figure 7A, the competition assay showed that the interaction of OmpL1 with the HMEC-1 cell line was reduced by 22 and 32% in the presence of 10 μg of collagen IV and laminin, respectively. In contrast, cellular fibronectin increased OmpL1 binding to HMEC-1 up to 63% (Figure 7A). For LipL41 and collagen IV, the interaction with HMEC-1 cells resulted in a decrease in binding, regardless of the concentration used (Figure 7B), while the incubation of the protein with laminin and cellular fibronectin increased binding by 55 and 800%, respectively (Figure 7B). In the case of the EA.hy926 cell line, both collagen I and collagen IV affected OmpL1 binding, producing reductions of 60 and 22%, respectively. The interaction with laminin also affected binding, reducing it by 37%. For that cell line, incubation with OmpL1 and cellular fibronectin increased binding, i.e., by 72% (Figure 7C). Interestingly, when the binding of LipL41 to EA.hy926 cells was assayed, collagen I, collagen IV, laminin, and cellular fibronectin produced increases in binding of 58, 100, 29, and 200%, respectively (Figure 7D). It is possible that a bridging effect may have occurred.

In summary, when OmpL1 was incubated with collagen IV and laminin, its binding to both HMEC-1 and EA.hy926 was impaired, suggesting a partial blocking of binding sites. As OmpL1 did not interact with collagen I and collagen IV [6], the reason its binding was reduced in EA.hy926 is unclear. Although LipL41 has been previously shown to interact with collagen I, collagen IV, laminin, and cellular fibronectin [10], its binding profile varied according to the cell line. When collagen IV was assayed with HMEC-1, reduced binding was observed. However, in the case of EA.hy926, the opposite effect was observed. Interestingly, cellular fibronectin increased binding of OmpL1 and LipL41 both for HMEC-1 and EA.hy926, suggesting a bridging effect, which could favor an increase in these interactions.

## 3. Discussion

The first step of the infection process in leptospirosis is pathogen invasion through the skin via cuts or abrasions, permeabilized skin in contact with contaminated water, and mucous membranes [23]. Leptospires access the bloodstream, circulate through the blood vessels and colonize target organs, such as kidney, liver, and lungs, which can cause hemorrhage and organ dysfunction [24]. Therefore, adhesion and invasion in different tissues (skin, endothelium, and kidney/lung/liver epithelium) is a crucial step for leptospires to maintain infection. However, the factors involved in these steps are still not completely elucidated.

Here, we investigated if the virulent, attenuated, and saprophytic strains of *Leptospira* and their major outer membrane proteins could bind to different mammalian cells, including endothelial and epithelial cells and fibroblasts. The first studies of leptospires adhesion to mammalian cells showed that virulent strains are more effective in binding to cells than are attenuated and saprophytic bacteria [4]. It was demonstrated that strains of *L. interrogans* serovars Canicola, Pomona, and Grippothyphosa interact with MDCK and PK-15 epithelial cells and HUVEC endothelial cells [17]. In another study, *L. interrogans* serovars Copenhageni and Canicola also showed robust binding to HEp-2, MDCK, and HMEC-1 cells [11]. Recently, an interesting study demonstrated that pathogenic *L. interrogans* can migrate through EA.hy926 and HMEC-1 monolayers, while *L. biflexa* cannot [25]. In the present study, we obtained similar results, where the virulent strain *L. interrogans* serovar Copenhageni bound strongly to EA.hy926, HMEC-1, and HULEC5a endothelial cells, to HEK293T and MDBK epithelial cells and to E. Derm and BHK-21 fibroblasts, more effectively than did the attenuated and saprophytic strains.

It is well-established that pathogenic leptospires have additional virulence factors, which are responsible for mediating interactions with host cells. However, the main bottleneck of leptospiral adhesion studies is functional redundancy of proteins, as observed in some mutagenesis experiments. For instance, the LigB mutant remained virulent and did not show differences in binding to MDCK cells between LigB knockout and wild-type *L. interrogans* [26]. So far, there are few leptospiral proteins assessed as binding factors for bacterial adhesion in mammalian cells. Here, we evaluated the binding potential of major leptospiral outer membrane proteins, OmpL37, OmpL1, LipL21, LipL41, and LipL46, to different mammalian cell lines, both in immobilized monolayers and detached cell suspensions. We showed that both OmpL1 and LipL41 display a wide profile of cell binding. We included OmpL1 as a positive control and confirmed its interaction with EA.hy926 endothelial cells [7]. LipL46 showed the ability to bind to HULEC5a endothelial cells and HEK293T and MDBK epithelial cells. Curiously, most proteins exhibited binding properties when evaluated in detached cell suspensions, suggesting interaction with cell receptors rather than ECM. Other proteins evaluated for their binding potential included the recombinant protein rLIC12976 to fibroblasts [20] and the adhesins rLIC10508, rLIC11574, rLIC13411, and rLIC12341 to EA.hy926 endothelial cells and HEp-2 cells [13], while rLIC10831 was found to bind to cadherins [18]. Other proteins evaluated that did not bind to cells were LipL32, Loa22, p31/LipL45, and LenA [7].

In an attempt to validate the specificity of these interactions, we used polyclonal antibodies against the recombinant proteins to evaluate the effect on the binding of virulent *L. interrogans* to mammalian cells. The use of antibodies to block specific binding sites and to prevent access to cell receptors has been observed in assays using the protein FbsA from *Streptococcus agalactiae* in A549 epithelial cells [27], the arg-gingipain A domain from *Porphyromonas gingivalis* in HEp-2 epithelial cells [28], the protein BmaC from *Brucella suis* in HeLa cells [29], the enteropathogenic *E. coli* flagella to HeLa cells [30], and the SEF17 fimbriae from *Salmonella enteritidis* in INT-407 epithelial cells [31]. Here, we observed significant inhibition of binding to the EA.hy926 and HEK293T cell lines after using anti-OmpL1 and anti-LipL41. Interestingly, the proteins OmpL37, LipL21, and LipL46, which also bind to the human kidney cell line HEK293T, showed a partial but statistically significant reduction in binding. Only the interaction of LipL41 with HMEC-1 cells had an opposite effect; where an increase rather than decrease in binding occurred. A similar behavior has already been described for OmpL37 protein, where an increase in bacterial adhesion to elastin was observed without bacterial agglutination [8]. Similar results were also obtained for other pathogens, such as the protein FnbA from *Streptococcus dysgalactiae*, which requires the presence of fibronectin for antibody recognition and stabilization of the protein-component binding [32], and the protein FnBPA from *Staphylococcus aureus* also showed an increase in protein–antibody ligation in the presence of fibronectin [33].

Assays using the recombinant proteins as a competitor factor in cell adhesion were also performed in an attempt to validate host–pathogen interactions. We observed a significant decrease in binding of *L. interrogans* to HMEC-1 and EA.hy926 only in the presence of OmpL1, and there was a decrease in binding with EA.hy926 in the presence of LipL41. OmpL37 caused a statistically significant reduction in bacterial binding to HEK293T. Although there was a trend towards reduced binding in the presence of other proteins, there was no significant difference. Despite our results, it was observed in *Leptospira* that the terminal regions containing immunoglobulin-like repeats from LigA and LigB were capable of inhibiting *L. interrogans* serovar Pomona adhesion to MDCK cells [21]. The use of the recombinant FbsA from *Streptococcus agalactiae* also inhibited adhesion to A549 epithelial cells [27]. Furthermore, the FBP54 protein from the Streptococcus group A was able to interact with fibronectin, and the co-incubation of the protein and bacteria in human buccal epithelial cells inhibited binding by 80% [34].

The use of host components was assessed to determine the interference of those components in protein binding to cells. We showed that laminin and collagen IV affected the binding of OmpL1 to HMEC-1 and EA.hy926 endothelial cells. Although it has been previously shown that OmpL1 does not interact with collagen IV [6], we observed that binding occurred at higher concentrations. The decrease in adhesion of OmpL1 using collagen IV and laminin is interesting, and this suggests that OmpL1 can bind to the same component binding sites or to the same cell receptor. It has also been shown for other pathogens, such as *Streptococcus*, *Staphylococcus*, *Yersinia*, *Treponema* and *Porphyromonas* [35]. In *Leptospira*, it was demonstrated for another component and the LigB protein. Thus, when LigB was co-incubated with tropoelastin, a decrease in adhesion to human pulmonary fibroblasts was observed [19]. In *Streptococcus pneumoniae*, its protein PsaA also showed a decrease in adhesion to nasopharyngeal epithelial cells when co-incubated with E-cadherin [36]. Regarding the LipL41 protein, its interaction with the components showed a different profile. However, for HMEC-1 cells, there was a decrease in binding after incubation with collagen IV, and the opposite effect was observed in EA.hy926. The reason for this occurring is unclear, but maybe LipL41 can have more than one collagen IV binding site. Thus, while the site is blocked in HMEC-1, in EA.hy926, collagen IV could bind to its cell receptor, acting as a bridge for LipL41. In the case of cellular fibronectin, an increase in binding to cells was observed for both OmpL1 and LipL41, suggesting that cellular fibronectin can be used as a helper molecule to allow leptospires to gain access to the host.

We conclude that the major outer membrane proteins OmpL37, OmpL1, LipL21, LipL41 and LipL46 of *Leptospira* can mediate bacterial adhesion to host cells. In particular, the work presented here offers evidence of direct binding of *L. interrogans* to several mammalian host cells and identifies OmpL1 and LipL41 as proteins with a broad cell binding profile in both endothelial and epithelial cells, and as potentially involved in this interaction. The elucidation of leptospiral adhesion is a crucial step in the development of new treatments to prevent host colonization. Thus, this study sheds new light on leptospiral adhesion and opens new windows for further investigation into the cell receptors involved. The use of antibodies against the cell receptors and leptospires knockout for OmpL1 and LipL41 will certainly contribute to these investigations.

## 4. Materials and Methods

### 4.1. Leptospira Strains and Mammalian Cell Lines

*L. interrogans* serovar Copenhageni (Fiocruz L1–130), *L. interrogans* serovar Copenhageni (M20), and *L. biflexa* serovar Patoc (Patoc 1), provided by the bacterial zoonosis laboratory of the Faculty of Veterinary and Animal Science of the University of Sao Paulo (USP), were grown in EMJH medium (Difco™, BD, Franklin Lakes, NJ, USA) supplemented with 10% Leptospira Enrichment EMJH (Difco™, BD). *L. interrogans* serovar Copenhageni (M20) has been culture-attenuated by passages since its isolation in 1938 from a patient in Denmark. Detailed information about the three strains used in this work can be found at the following website: (https://leptospira.amsterdamumc.org/leptospirosis-reference-centre/, accessed on 28 September 2020). All human cells were purchased from ATCC (ATCC, Manassas, VA, USA). The human endothelial cell lines HMEC-1 (dermal microvascular) and HULEC5a (lung microvascular) were grown in MCDB 131 medium (M8537, Sigma-Aldrich, St. Louis, MO, USA) supplemented with 20% fetal bovine serum (FBS, Bionutrientes, São Paulo, SP, Brazil), 10 ng/mL epidermal growth factor (E9644, Sigma-Aldrich), 1 µg/mL hydrocortisone (H0888, Sigma-Aldrich), and 10 mM glutamine (ATCC). The cell line EA.hy926 (endothelial macrovascular somatic cell hybrid) and HEK293T (epithelial cells from kidney) were grown in Dulbecco’s Modified Eagle Medium (DMEM, D7777, Sigma-Aldrich) with 4.5 g/L glucose and supplemented with 10% FBS and 2 mM glutamine. The mammalian cells E. Derm (fibroblasts from horse skin), BHK-21 (fibroblasts from hamster kidney) and MDBK (epithelial cells from bovine kidney) were purchased from Adolfo Lutz Institute (IAL, São Paulo, SP, Brazil) and were grown in RPMI 1640 (Roswell Park Memorial Institute 1640 medium, Gibco, Grand Island, NY, USA) supplemented with 10% FBS and 2 mM glutamine. The cell lines were grown at 37 °C under 5% CO_2_ and tested for mycoplasma contamination before the assays.

### 4.2. Evaluation of Leptospire Binding to Mammalian Cells by ELISA

Ninety-six-well cell culture plates (3599, Corning Costar, Corning, NY, USA) were previously incubated with 125 µL of 0.01% poly-L-lysine (P8920, Sigma-Aldrich) in phosphate-buffered saline (PBS) for 5 min. The solution was aseptically removed, and the plates were dried at 37 °C for 1 h. Mammalian cells (10^5^ cells/well) were seeded and incubated at 37 °C under 5% CO_2_ for 24 h to achieve maximum confluence. Next, 10^7^ leptospires (MOI 100) were resuspended in cell medium without supplements and were added at 37 °C under 5% CO_2_ for 2 h. Wells without addition of bacteria were used as negative control. Plates were washed three times with PBS containing 0.05% Tween 20 (PBS-T) to remove unbound bacteria. Leptospires bound to cells were fixed with 2% paraformaldehyde in PBS at 25 °C for 30 min. Plates were washed, and the wells were incubated with polyclonal anti-*Leptospira* antibody produced in hamster against each strain (1:1000) at 37 °C for 1 h. Detection of bound bacteria was performed using HRP-conjugated anti-hamster IgG (1:5000) for 1 h, followed by the addition of citrate phosphate buffer (150 mM, pH 5.0) containing 1 mg/mL *o*-phenylenediamine (P8287, Sigma-Aldrich) and 0.03% H_2_O_2_. After 15 min, the reaction was stopped by adding 2 M H_2_SO_4_, and the absorbance (A 492 nm) was read at 492 nm using a Multiskan-FC microplate reader (Thermo Fisher Scientific, Helsinki, Finland).

### 4.3. Determination of Leptospiral Membrane Protein Binding to Mammalian Cells

Recombinant proteins were obtained by cloning the gene sequences of *OmpL37*, *OmpL1*, *LipL21*, *LipL41*, and *LipL46* without the signal peptide into the pET-SUMO vector (Table S1). The proteins were expressed in an *E. coli* system and purified by immobilized metal affinity chromatography, as previously described [10]. The presence of surface proteins was previously evaluated by ELISA. The culture plates were incubated with poly-L-lysine as previously described when HEK293T and BHK-21 cells were seeded. All cell lines were seeded in 96-well culture plates (10^5^ cells/well) and were incubated at 37 °C under 5% CO_2_ for 24 h to achieve maximum confluence. Wells were incubated with 1 µg of each recombinant protein and 1 µM aprotinin (A1153, Sigma-Aldrich) resuspended in culture medium without supplementation at 37 °C under 5% CO_2_ for 2 h. The final concentration of each recombinant protein was 281 nM for OmpL37, 317 nM for OmpL1, 549 nM for LipL21, 268 nM for LipL41, and 232 nM for LipL46. Wells without addition of proteins were used as negative control. Plates were washed three times with PBS-T, and bound bacteria were fixed with 2% paraformaldehyde at 25 °C for 30 min. Plates were incubated with polyclonal antibodies produced in mouse against each protein (dilution of 1:1000 for OmpL37, OmpL1, LipL41, and LipL46 and 1:500 for LipL21) at 37 °C for 1 h. After the washing step, the plates were incubated with HRP-conjugated anti-mouse IgG (1:5000) at 37 °C for 1 h. Detection was performed as previously described. The specificity of binding was assessed by dose-dependent effect with increase in protein concentration. Binding of the recombinant proteins was also assessed with cell suspension incubation. Cell culture monolayers in T75 flasks were trypsinized, and cells resuspended with culture medium without supplementation (2 × 10^5^ cells/sample). Recombinant proteins were added at three different concentrations containing 1 µM aprotinin to a final volume of 200 µL and incubated at 37 °C under 5% CO_2_ for 2 h. Cells were centrifuged at 130× *g* for 5 min. Supernatant was removed, and the pellet was resuspended in 200 µL of PBS. Protein was detected from supernatant and pellet samples by Western blot by using polyclonal antibodies produced in mouse against each protein as described above. The purified recombinant protein was used as positive control.

### 4.4. Effect of Leptospiral Membrane Proteins on Bacterial Adhesion to Mammalian Cells

Cell culture plates were pre-incubated with poly-L-lysine, and 10^5^ cells/well were seeded to form confluent cell monolayers. Leptospires (10^7^ bacteria/well, MOI 100) were incubated with polyclonal antibodies against the membrane proteins (1:100) at 37 °C for 1 h. The samples were then added to plates at 37 °C under 5% CO_2_ for 2 h. Wells without added bacteria and samples without added polyclonal antibodies were used as controls, representing 0 and 100% binding, respectively. Bound leptospires were fixed with 2% paraformaldehyde at 25 °C for 30 min. Detection of leptospires was performed using polyclonal antibodies produced in hamster against LipL32 (1:1000), followed by incubation with HRP-conjugated anti-hamster IgG (1:5000). The detection reaction and reading were performed as previously described.

The second method was performed with the incubation of the recombinant proteins in cell culture plates before the addition of leptospires. Recombinant proteins were added at the saturation concentration for each cell line with 1 µM aprotinin at 37 °C under 5% CO_2_ for 1 h (Table 2). Bound leptospires were fixed with 2% paraformaldehyde at 25 °C for 30 min. Leptospires were added (MOI 100) at 37 °C for 2 h. Wells without added bacteria and samples without added recombinant proteins were used as controls, representing 0 and 100% of binding, respectively. Bound leptospires were fixed with 2% paraformaldehyde at 25 °C for 30 min. Detection of leptospires was performed using polyclonal antibodies produced in mouse against LipL32 (1:1000), followed by incubation with HRP-conjugated anti-mouse IgG (1:5000). The detection reaction and reading were performed as previously described.

### 4.5. Evaluation of the Effect of Host Components on Adhesion of Leptospiral Membrane Proteins to Mammalian Cells

Cell culture plates were seeded with 10^5^ cells/well and incubated at 37 °C under 5% CO_2_ for 24 h. Recombinant proteins were incubated with different concentrations of host components (Table 3, collagen I–C3867, collagen IV–C7521, laminin–L2020, and cellular fibronectin–F2518, Sigma-Aldrich) and 1 µM aprotinin in cell culture medium without supplementation at 37 °C for 1 h. Samples without protein and without added host components were used as controls, representing 0% adhesion. Samples with protein but without added host components were used as 100% adhesion. Cells were incubated with protein samples at 37 °C under 5% CO_2_ for 2 h. Bound bacteria were fixed with 2% paraformaldehyde at 25 °C for 30 min. Detection of recombinant protein was performed using polyclonal antibodies produced in mouse against each protein (1:1000), followed by incubation with HRP-conjugated anti-mouse IgG (1:5000). The detection reaction and reading were performed as previously described.

### 4.6. Statistical Analysis

The evaluation of statistical significance between the controls and experimental groups were performed by two-tailed *t*-test (*p* < 0.05) in GraphPad Prism software v. 7 (GraphPad Prism, San Diego, CA, USA). In the figures, we only indicated the significance for the experimental groups. The dose-response curves and dissociation constant calculation were fitted by the tool in GraphPad Prism software v. 7 “Non-linear regression”, considering saturation binding setting “one site-total”.

The blocking assays were compared with the samples represented by 100% of binding, i.e., the wells treated with bacteria or recombinant protein without incubation with blocking factors. The values represented by percentage were normalized by GraphPad Prism v. 7. The basal value of 0% was assumed as the average of the wells without bacteria or recombinant protein. The 100% of adhesion was assumed as the average of the wells treated with leptospires or recombinant protein without the incubation with blocking factors. The inhibition of adhesion was expressed by the subtraction of 100% from the normalized values.

## Figures and Tables

**Table 1:** Dissociation constants (K_D_) represented in nM from protein binding to immobilized mammalian cells.

*Protein*	Mammalian Endothelial Cells		
	*EA.hy926*	*HMEC-1*	*HULEC5a*
*OmpL1*	40.8 ± 11.9	174.8 ± 136.1	55.2 ± 54.5
*LipL41*	-	11.7 ± 6.0	-
*LipL46*	-	-	-
***Protein***	**Mammalian Epithelial Cells**		
	***HEK293T***	***MDBK***	
*OmpL37*	606.5 ± 223.4	-	
*OmpL1*	-	40.9 ± 19.4	
*LipL21*	28.7 ± 14.5	-	
*LipL41*	280.1 ± 108.8	53.2 ± 34.2	
*LipL46*	-	1576 ± 1325	
***Protein***	**Mammalian Fibroblasts**		
	***E. Derm***	***BHK-21***	
*OmpL1*	20.6 ± 9.9	-	
*LipL41*	3.6 ± 2.6	-	

**Table 2:** Recombinant protein concentration (nM) and weight (μg) added to leptospire blocking assay in cell monolayers.

Protein	Cell Line					
	HMEC		EA.hy926		HEK293T	
	(nM)	(μg)	(nM)	(μg)	(nM)	(μg)
OmpL37	4070	17.5	507	2.2	507	2.2
OmpL1	793	2.5	198	0.6	198	0.6
LipL21	2750	5.0	343	0.6	343	0.6
LipL41	672	2.5	1340	5.0	672	2.5
LipL46	8140	35.0	8140	35.0	8140	35.0

**Table 3:** Host components, with molecular mass (kDa), concentration (nM) and weight (μg), added to recombinant protein blocking assay using cell monolayers.

Host Component	Molecular Weight	Concentration (nM)		
	(kDa)	1 μg	5 μg	10 μg
Collagen I	695	14.4	71.9	143.8
Collagen IV	492.5	20.3	101.5	203.1
Laminin	810	12.4	61.7	123.5
Cellular fibronectin	550	18.2	90.9	181.8

## Supplementary Materials

The following supporting information can be downloaded at: https://www.mdpi.com/article/10.3390/ijms232415550/s1.
