# Supplementary material for: Host Cell Binding Mediated by Leptospira interrogans Adhesins
Source: Int J Mol Sci. 2022 Dec 8;23(24):15550. doi: 10.3390/ijms232415550 (PMC9779477; doi:10.3390/ijms232415550)
Supplement: Supplementary file 1 [file ijms-23-15550-s001.zip › ijms-1979197-supplementary.pdf]

Figure S1. 12% SDS-PAGE analysis of the recombinant proteins. The proteins OmpL1, OmpL37, LipL21, LipL41 and LipL46 were expressed in *Escherichia coli* using the pET-SUMO vector. After purification using immobilized metal affinity chromatography, the fusion proteins were cleaved using the enzyme Ulp-1. Proteins were isolated from the SUMO tag and Ulp-1 by a second chromatographic step. Lanes were represented by M: molecular mass marker, 1: OmpL1, 2: OmpL37, 3: LipL21, 4: LipL46 and 5: LipL41.

Figure S2. The effect on the binding of increasing concentration of recombinant proteins to endothelial cell monolayers. Ninety-six-well plate were seeded with 10^5 cell/well at 37°C under 5% CO_2 for 24 h. Proteins were added in cell media with 1 µM aprotinin for 2 h. Binding was fixed with 2% paraformaldehyde for 30 min at room temperature. Polyclonal antibodies against the recombinant proteins (1:5,000) were added for 1 h. Reaction was detected with anti-mouse IgG-peroxidase (1:5,000). The dose-dependent curves were fitted using the GraphPad Prism software. Bars and points represent the mean absorbance at 492 nm ± SD of three replicates. A: EA.hy926 and OmpL1, B: HMEC-1 and OmpL1, C: HMEC-1 and LipL41, D: HULEC5a and OmpL1, E: HULEC5a and LipL46 and F: EA.hy926 and LipL41.

Figure S3. The effect on the binding of increasing concentration of recombinant proteins to epithelial cell monolayers. Ninety-six-well plate were seeded with 10^5 cell/well at 37°C under 5% CO_2 for 24 h. Proteins were added in cell media with 1 µM aprotinin for 2 h. Binding was fixed with 2% paraformaldehyde for 30 min at room temperature. Polyclonal antibodies against the recombinant proteins (1:5,000) were added for 1 h. Reaction was detected with anti-mouse IgG-peroxidase (1:5,000). The

dose-dependent curves were fitted using the GraphPad Prism software. Bars and points represent the mean absorbance at 492 nm ± SD of three replicates. A: HEK293T and OmpL37, B: HEK293T and OmpL1, C: HEK293T and LipL21, D: HEK293T and LipL41, E: HEK293T and LipL46, F: MDBK and OmpL1, G: MDBK and LipL41 and H: MDBK and LipL46.

Figure S4. The effect on the binding of increasing concentration of recombinant proteins to fibroblast cell monolayers. Ninety-six-well plate were seeded with 10^5 cell/well at 37°C under 5% CO_2 for 24 h. Proteins were added in cell media with 1 µM aprotinin for 2 h. Binding was fixed with 2% paraformaldehyde for 30 min at room temperature. Polyclonal anti-bodies against the recombinant proteins (1:5,000) were added for 1 h. Reaction was detected with anti-mouse IgG-peroxidase (1:5,000). The dose-dependent curves were fitted using the GraphPad Prism software. Bars and points represent the mean absorbance at 492 nm ± SD of three replicates. A: E. Derm and OmpL1, B: E. Derm and LipL41, C: BHK-21 and OmpL1, and D: BHK-21 and LipL41.

Figure S1

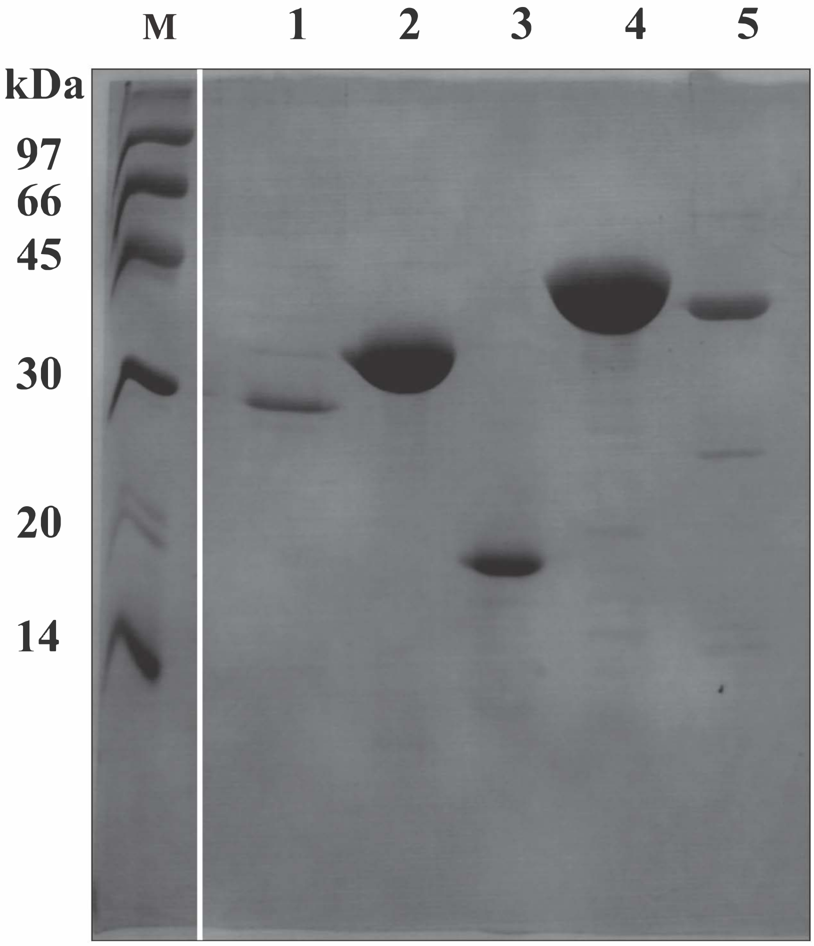

Figure S2

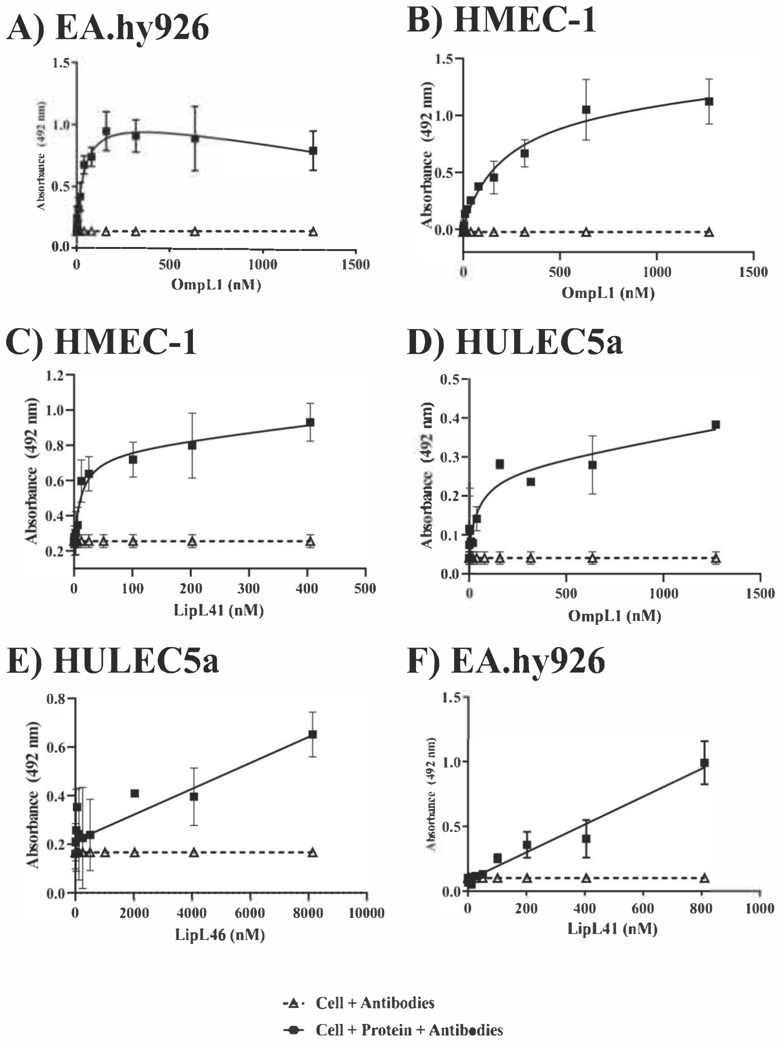
A) EA.hy926
Absorbance (492 nm)
OmpL1 (nM)
B) HMEC-1
Absorbance (492 nm)
OmpL1 (nM)
C) HMEC-1
Absorbance (492 nm)
LipL41 (nM)
D) HULEC5a
Absorbance (492 nm)
OmpL1 (nM)
E) HULEC5a
Absorbance (492 nm)
LipL46 (nM)
F) EA.hy926
Absorbance (492 nm)
LipL41 (nM)
Cell + Antibodies
Cell + Protein + Antibodies

Figure S3

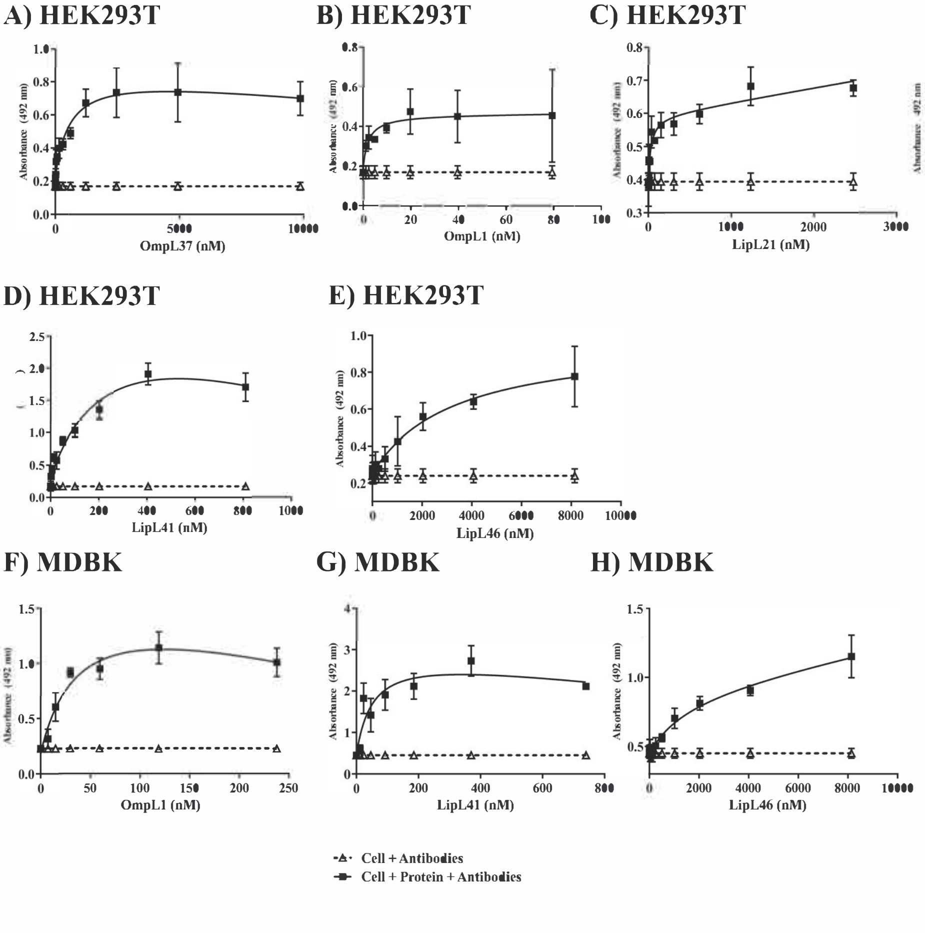

A) HEK293T
B) HEK293T
C) HEK293T
D) HEK293T
E) HEK293T
F) MDBK
G) MDBK
H) MDBK
Absorbance (492 nm)
OmpL37 (nM)
OmpL1 (nM)
LipL21 (nM)
LipL41 (nM)
LipL46 (nM)
Cell + Antibodies
Cell + Protein + Antibodies

Figure S4

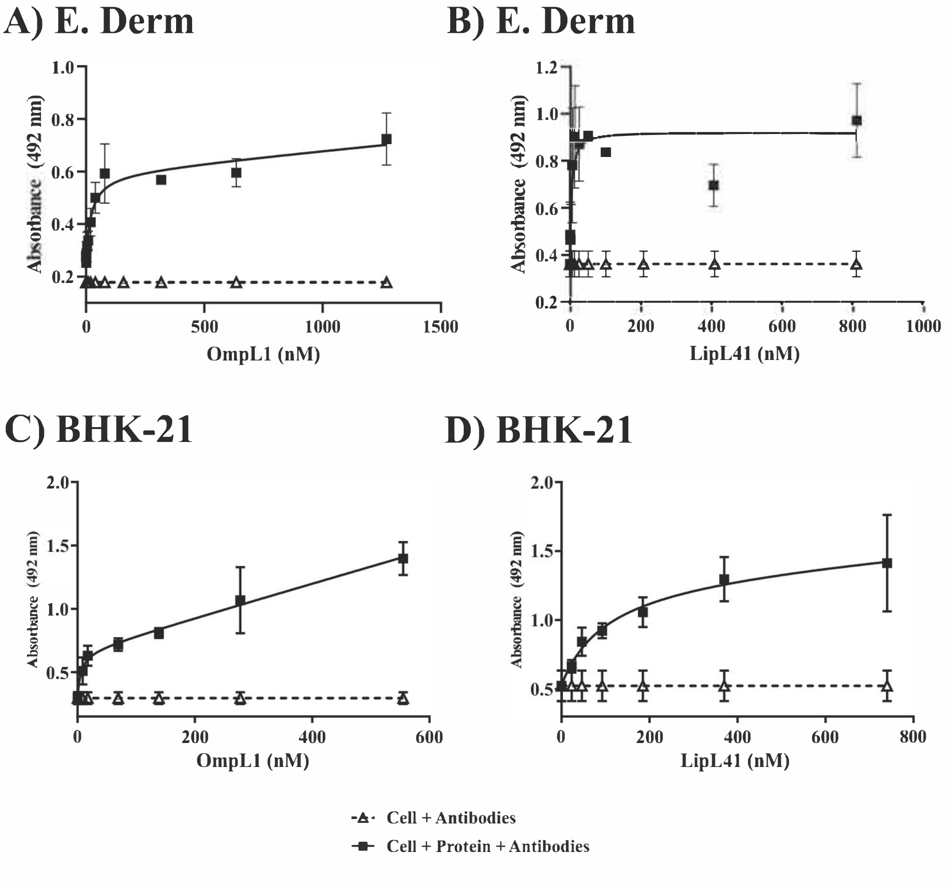
A) E. Derm
B) E. Derm
C) BHK-21
D) BHK-21
Absorbance (492 nm)
OmpL1 (nM)
LipL41 (nM)
Cell + Antibodies
Cell + Protein + Antibodies

Table S1. Primers used to amplify the genes of interest

Gene	Sequences
LipL21	*Forward* 5’ CGC **GAATTC** TCCAGTACTGACACAGGA 3’ (*Eco*RI)
	Reverse 5’ GGCG **CTCGAG** TTATTGTTTGGAAACCTC 3’ (*Xho*I)
LipL41	*Forward* 5’ GCG **GAATTC** GCAGCTACAGTCGATGTAGAA 3’ (*Eco*RI)
	Reverse 5’ CGCG **CTCGAG** TTACTTTGCGTTGCTTTCATC 3’ (*Xho*I)
LipL46	*Forward* 5’ATT **GAGCTC** GGTTCTTCCGGTTCCACT 3’ (*Sac*I)
	Reverse 5’ CGGC **GTCGAC** TTATTTCAAAGGTTTGAA 3’ (*Sal*I)
OmpL1	*Forward* 5’GCCG **GAATTC** ATCACCAAAGATGGTTTA 3’ (*Eco*RI)
	Reverse 5’ CGCG **CTCGAG** TTAGAGTTCGTGTTTATA 3’ (*Xho*I)
OmpL37	*Forward* 5’CTG **GAATTC** GTTTCGCCGGATCAGATC 3’ (*Eco*RI)
	Reverse 5’ GCGCC **CTCGAG** TTAATTTTGTGTTTTTGT 3’ (*Xho*I)
